# The Role of Primary Cilia in Thyroid Cancer: From Basic Research to Clinical Applications

**DOI:** 10.3389/fendo.2021.685228

**Published:** 2021-06-08

**Authors:** Cheng-Xu Ma, Xiao-Ni Ma, Ying-Dong Li, Song-Bo Fu

**Affiliations:** ^1^ Department of Endocrinology, The First Hospital of Lanzhou University, Lanzhou, China; ^2^ The First Clinical Medical College of Lanzhou University, Lanzhou, China; ^3^ College of Integrated Traditional Chinese and Western Medicine, Gansu University of Chinese Medicine, Lanzhou, China

**Keywords:** primary cilium, thyroid cancer, cell cycle, ciliogenesis, therapeutic strategy

## Abstract

Primary cilia (PC) are microtubule-based organelles that are present on nearly all thyroid follicle cells and play an important role in physiological development and in maintaining the dynamic homeostasis of thyroid follicles. PC are generally lost in many thyroid cancers (TCs), and this loss has been linked to the malignant transformation of thyrocytes, which is regulated by PC-mediated signaling reciprocity between the stroma and cancer cells. Restoring PC on TC cells is a possible promising therapeutic strategy, and the therapeutic response and prognosis of TC are associated with the presence or absence of PC. This review mainly discusses the role of PC in the normal thyroid and TC as well as their potential clinical utility.

## Introduction

Primary cilia (PC) are solitary, nonmotile microtubule-based organelles that project from the cell surface and function similar to an antenna on the cell to sense and convey multiple extracellular signals (1). In thyroid follicular epithelial cells, PC protrude from the apical surface into the follicular luminal space, where they may sense the follicular luminal environment and transmit signals to follicular epithelial cells to maintain follicular homeostasis (2). Existing data have revealed that many signaling pathways important for development and disease, including the Hedgehog (Hh), Wnt, and platelet-derived growth factor (PDGF) pathways, are localized to PC (3). Therefore, a loss of PC is associated with the onset of malignancy in some human tumors.

Thyroid cancer (TC) is the most common endocrine cancer and has a rapidly increasing incidence but relatively stable mortality. The main histological subtypes of TC are papillary thyroid cancer (PTC), follicular thyroid cancer (FTC), poorly differentiated thyroid cancer (PDTC), anaplastic thyroid cancer (ATC), and medullary thyroid cancer (MTC). The first four types originate from thyroid follicular epithelial cells, and MTC arises from thyroid parafollicular cells. PC is well preserved in PTC and FTC, and their frequency and length appear similar to those of normal thyroid follicles. Interestingly, defects in PC genesis have been observed in ATC (4). Additionally, oncogenic alterations, coupled to specific intracellular downstream signaling pathways, lead to the development of different subtypes of TC. PC as a mediator of these signaling pathways regulates TC development. Alteration in PC influences the communication between TC cells and the tumor microenvironment, which in turn affected the therapeutic response and prognosis of TC.

In this review, we briefly describe the formation and structure of PC on thyroid follicular cells and explore the potential roles of PC in maintaining cellular homeostasis and promoting the progression of thyroid disease.

## Structure of PC in the Normal Human Thyroid Gland

PC consist of the basal body, transition zone and axoneme. The basal body derived from the mother centriole of the centrosome that is composed of nine microtubule triplets (5). The axoneme is constructed from nine parallel microtubule doublets protruding from the mother centriole, which anchors the PC within the plasma membrane. The transition zone is a specialized ciliary domain that connects the basal body and axoneme backbone, which is localized toward the membrane in a Y-shaped arrangement known as the “ciliary gate.” This gate separates proteins inside the PC from proteins in the cytoplasm and limits extracellular signal transduction.

In human thyroid follicular cells, PC are present in a ring-shaped 9 + 0 axonemal configuration, and the microtubules and diameters of PC steadily decrease toward the distal end of the cilia while becoming broader closer to the base of the cilium. Dynein arms and central pair microtubules are absent (6). Therefore, PC are non-motile structures. The length of PC ranges between 5.0 and 10.7 μm, and the mean length is 7.3 ± 1.2 μm (7). Almost all human thyroid follicular cells displays at least one PC that protrudes from the apical surface into the follicular lumen, and occasionally, the presence of two PC in a V-shaped distribution has been observed on adjacent cells. The PC of thyroid follicular cells taking advantage of their ideal localization coupled to specific intracellular downstream signaling pathways regulates thyroid development.

## Abnormal Length and Frequency of PC in TC

The frequency and length of PC often change when they respond to diverse stimuli from both inside and outside of thyrocytes. In TC, the distribution and frequency of PC are aberrantly changed, the number of thyrocytes exhibiting one or more cilia steadily decreases from ordinary PTC to FTC, and FTC, PDTC, and ATC usually lack cilia (8). However, conventional PTC and follicular variants of PTC display well-expressed PC, the length of PC is strikingly increased, and the frequency of PC seems to be unchanged compared with normal thyroid glands, only oncocytic variants of PTC have a decreased frequency and length of PC (9). More importantly, these variations of PC appear to be associated with the progression and prognosis of TC. Moreover, the pathogenicity of PC variations in the experimental animals of thyroid gland was also observed. A mouse model lacking PC showed normal folliculogenesis and hormonogenesis at ages of less than 7 weeks. After that, thyroid follicles became irregularly dilated and destroyed and displayed malignant properties (papillary or solid proliferative nodules), papillary or solid hyperplastic nodules were considered PDTC (2), and other types of TC were not observed.

In cell level, the frequency of PC is not different between human normal thyroid follicular cells and PTC cell lines, but the frequency of PC is significantly reduced in ATC cell lines (2). The loss of PC on ATC cells was associated with TC tumorigenesis and progression (10). However, a study from Junguee Lee et al. demonstrated that loss of PC in PTCs results in increased apoptosis, and it is associated with reduced tumor aggressiveness (4). In this context, the loss of PC appears to select TC cells with more malignant features. These discrepant conclusions require further confirmation due to the use of different research models.

**Figure 1:**
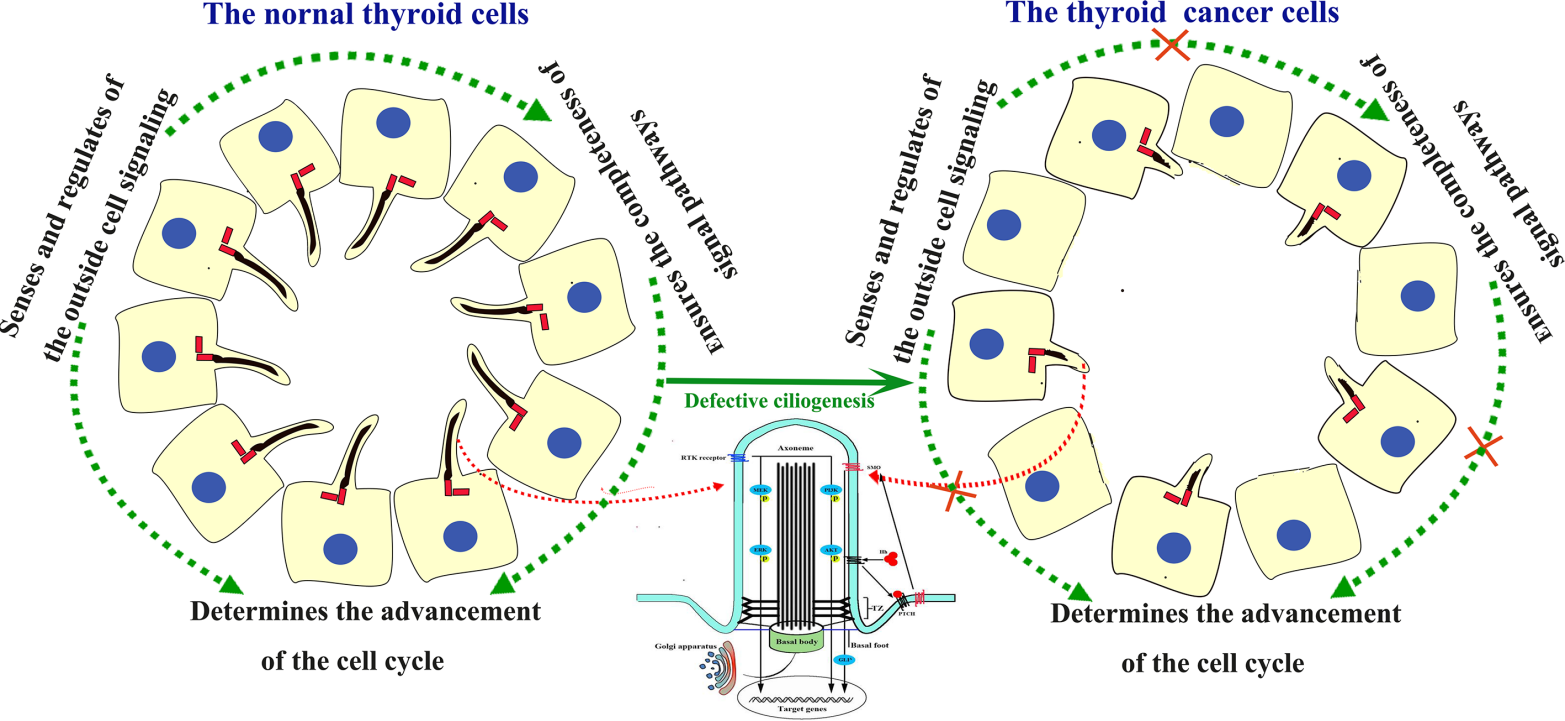
Structure of the PC and signaling pathways involving cilia in the human thyroid gland and TC. In normal thyroid gland cells, the PC acts as the sensor and regulator of the outside cell signaling. In TC cells, the PC was absent. Absence of the PC block the signal from outside and neighbors, and disturb the signal pathways inside cells, thus lead to uncontrolled cell cycle process and tumorigenesis.

In conclusion, some possible mechanisms for the association with PC abnormal changes and TC tumorigenesis have been proposed (**Figure 1**). So the loss of PC is at least partially associated with the tumorigenesis and progression of TC.

## PC Mediate HH, MAPK, and PI3K Signaling in TC

Some signaling pathways related to the occurrence, development, invasion, and metastasis of TC are mediated by PC, and the receptors for these signaling pathways are often localized to PC. In the presence of Hh, Ptch1 is transported out of the cilium, and Smo is transported into the cilium, where it promotes the formation of the activator form of Gli. Gli protein levels increase in the cilium, and Gli proteins are then transported out of the cilium and into the nucleus, where they activate Hh target genes. In the absence of Hh, Ptch1 is localized to the ciliary membrane, Smo is excluded from the cilium, and Gli is converted to its repressor form (11). At an early phase of TC development, TC stroma secreted Hh ligand mediates tumor-stroma interaction and Hh pathway are aberrantly activated, which supports TC cell invasion, migration, and growth in non-adherent conditions (12). Additionally, PC provide a spatially diverse platform for mediating interaction between the stroma and cancer cells, so PC genesis defects may disturb this interaction mediating Hh pathway aberrant activation. These data show that the PC-mediated Hh pathway participates in TC tumorigenesis.

On the other hand, PC-mediated growth factor binding to RTKs triggers the MAPK and PI3K-AKT cascades to regulate TC cell proliferation, and elevated RTK activity also promotes *RET/RAS/BRAF* mutations (13, 14). Moreover, oncogenic RAS/BRAF/MEK pathway influences Hh pathway activation in TC cell, generating a ligand independent non-canonical mechanism of activation.

Since PC are the carrier of these signaling proteins, and they integrate Hh and RTK signaling crosstalk to coordinate thyroid hormone synthesis and development. Changes of PC directly affect these related pathways, which is associated with tumorigenesis and TC development.

## PC Protrusion and Resorption Affect TC Cell Fate

PC protrusion and resorption are tightly coordinated with the cell cycle; in addition, the coupling of both ciliogenesis and the cell cycle depends on the centrosome, and the role of the centrosome shifts from cell division to ciliogenesis (15). Cells become quiescent, or proliferating cells enter G1 phase (16), the centrioles from the centrosome begin to migrate to the cell surface and subsequently form the basal body, and assembly of PC is initiated; the PC then extend from the basal body into the follicular lumen. In dividing cells, PC are resorbed before S phase or during G2 phase (17). Subsequently, the centrosome forms the mitotic spindle, and the cell enters mitosis. After mitosis, centrosomes are again available to assemble PC, either in G0 or in early G1 phase (18). Utrilla confirmed this process in thyroid cells (7). These findings suggest that ciliogenesis and the cell cycle appear to regulate each other *via* mutual crosstalk.

## PC Proteins Affect TC Progression

PC proteins related to Aurora kinase A, NIMA-related kinases (Nek), Polo-like kinase (Plk1), and spermatogenesis-associated protein 4 (Spata4) have been identified as critical for the regulation of both cilia and the cell cycle. Aurora kinase A, which is localized to the centrosome and radial microtubules in PC (19), is highly or weakly expressed in TC tissues and cell lines, and this abnormal expression induces thyrocyte malignant transformation. Moreover, activation of Aurora kinase A in late G2 phase triggers spindle assembly and PC disassembly, and it becomes inactivated at the completion of mitosis. Overexpression of Aurora kinase A generally implies a poor prognosis and is a new molecular target in TC therapy (20). Nek, which is localized to PC and centrosomes, inhibits ciliogenesis and promotes spindle assembly, and it may contribute to the coordination between ciliogenesis and cell cycle progression (21). Overexpression of Nek 1 in classical and follicular variants of PTC has a high specificity and sensitivity and is often related to aggressiveness. Therefore, Nek 1 may contribute to the identification of malignant features during TC diagnosis (22). Plk1, which is localized to the transition zone of PC, rapidly evokes PC resorption and regulates cell cycle progression at the G2-M phase transition (23, 24). Plk1 expression is only occasionally observed in normal thyrocytes, but overexpression of Plk1 is more frequently detected in smaller PTCs, microcarcinomas, and incidental carcinomas. Overexpression of Plk1 may be an early event in PTC progression (25). Spata4 is a spermatogenesis-associated protein associated with thyroid hormone in fish (26), and spata4 knockdown leads to an arrest of cells in S phase and causes a decrease in the number of cilia in human retinal epithelial cells (27). The orderly regulation of these PC proteins between ciliogenesis and the cell cycle determines the survival of TC cells.

We focused on how PC proteins are linked to cell cycle progression and ciliogenesis in TC. Although the mechanisms by which TC cells lose their cilia are unknown, Aurora kinase A, Nek, Plk1, and Spata4 are associated with cilium disassembly and cell cycle regulation.

## Drugs With Effects on PC

The effects of drugs on PC in human cell lines and experimental animal models of TC have not been identified, but U0126 inhibits the elongation of cilia, ganetespib causes the loss of PC in experimental animal models of other diseases (28, 29). Additionally, the administration of U0126 and ganetespib effectively inhibits the proliferation of different histological types of TC cells (30, 31). Evidence for the association of U0126 and ganetespib with PC should be investigated in TC.

Drugs targeting PC have been assessed in patients with TC, and the findings from clinical trials show that doxorubicin, paclitaxel and docetaxel have modest antitumor activity in patients with advanced, nonmedullary TC (32). Carboplatin is recommended as a treatment for ATC in clinical practice guidelines in oncology (33). The administration of these drugs for TC may provide a clinical benefit in adjuvant settings. Doxorubicin induces cilium formation in breast fibroblasts (34), paclitaxel causes cilium elongation in the quail oviduct (35), and docetaxel decreases cilia numbers in olfactory cells (36). In addition, carboplatin induces PC disassembly in sensory cells (37) and has been used to treat TC. Some RTK inhibitors have been recently approved for use in clinical practice, namely sorafenib and lenvatinib, have been approved for differentiated thyroid cancer (DTC) and PDTC, and vandetanib and cabozantinib have been approved for MTC (38), but their effect on PC has not been reported.

Anticancer drugs with documented effects on PC have been proposed. These drugs induce PC disassembly, restore ciliogenesis, lengthen PC, and prevent the accumulation of Smo in PC. They have been assessed in TC in preclinical and clinical studies, considering these factors will formulate rational therapeutic strategies for TC.

## Is Restoring Cilia a Promising Therapeutic Strategy in TC Cells?

Although controversy exists regarding whether the length of PC is modified in PTC, the frequency of PC in TC cells is usually decreased. In addition, ATC and MTC exhibit loss of PC, and dysfunction of PC in TC cells is associated with tumorigenesis and malignancy. More importantly, in Hh pathway-dependent cancers, there is loss of PC as a mechanism of resistance to Smo inhibitors (39). Accordingly, restoring cilia may be a promising therapeutic strategy in TC (40).

There have been some pre-clinical examples of restoration of PC as a therapeutic target, such as restoration of PC by HDAC inhibitors in cholangiocarcinoma reduce cholangiocarcinoma cell growth (41–43). In addition, the use of HDAC inhibitors to treat TC has been well described, although they failed to trigger a major response against PTC, ATC, and MTC in clinical trials, HDAC inhibitors produced encouraging results in PTC, ATC, and MTC cell lines (44–46). Compounds (gefitinib and dexamethasone) that are able to restore cilia have been verified in cell lines of multiple human cancers (such as pancreatic, kidney, breast, and lung cancers) (47). Gefitinib shows limited effectiveness in patients with advanced TC (48), and dexamethasone exerts antiproliferative effects on a human MTC cell line (49).

It is hard to say that the restoring of cilia by those drugs was a primary effect or a secondary effect. However, several drugs with effects on PC have been tested in different human cell lines of TC, which indicate a possible application of these drugs in clinical studies. Future studies are required to evaluate the effects of these drugs on PC of TC.

## Conclusions and Future Prospects

Based on the findings described above, PC play a role in sustaining thyroid follicular cell polarity, differentiation, and proliferation and feature signaling pathways associated with TC. PC undergo cycles of assembly and disassembly that control TC cell survival, and PC loss in TC cells is usually linked to tumor aggressiveness in the clinic. PC in thyroid cells appear to function as tumor suppressors, and treatments that restore PC may be a potentially promising therapeutic strategy for TC. Preclinical and clinical studies will be required to test the roles of PC in the occurrence and progression of TC.

In the future, the relationship between ciliogenesis and pathological differentiation of TC tissue should receive increasing attention. Studies of the effects of potential oncogenic genetic mutations on PC formation may further reveal the mechanisms underlying TC pathogenesis, and powerful omics analyses have the potential to provide key insights into the role of PC in TC. Therefore, larger studies are required to assess whether the absence of PC drives TC transformation or is a secondary effect of tumorigenesis, which will be critical when considering ciliotherapy as a potential strategy.

## Author Contributions

C-XM, X-NM, Y-dL, and S-BF conceived the study and wrote the paper. All authors contributed to the article and approved the submitted version.

## Funding

The author(s) disclose receipt of the following forms of financial support for the research, authorship, and publication of this article: this work was supported by grants from the Construction Program of Gansu Provincial Clinical Medical Research Center for Endocrine Diseases (20JR10FA667), The Gansu Provincial Natural Science Foundation (20JR10RA681), The Lanzhou Chengguan District Science and Technology Plan Project (2019SHFZ0038), and The Special Funds of Science and Technology Development of the Chinese Central Government to guide Local in 2020 (1004TCYA032).

## Conflict of Interest

The authors declare that the research was conducted in the absence of any commercial or financial relationships that could be construed as a potential conflict of interest.
